# Supplementary material for: Extracellular matrix abnormalities in the hippocampus of subjects with substance use disorder
Source: Transl Psychiatry. 2024 Feb 24;14:115. doi: 10.1038/s41398-024-02833-y (PMC10894211; doi:10.1038/s41398-024-02833-y)
Supplement: Supplementary file 1 — Supplemental Materials [file 41398_2024_2833_MOESM1_ESM.pdf]

Supplemental Materials

Human subjects used for brightfield microscopy and RNA studies

All procedures were approved by the Institutional Review Boards of the University of Mississippi Medical Center, Jackson, MS and the University Hospitals Cleveland Medical Center, Cleveland, OH, and are in accordance with the Declaration of Helsinki. Informed consent from the legally-defined next-of-kin was obtained for the collection of tissue, medical records, and retrospective psychiatric interviews. Structured Clinical Interview for DSM-IV Axis I Disorders was administered by a Master-level social worker to knowledgeable informants of the subjects, as outlined in (1). To determine the subjects' psychopathology, a board-certified clinical psychologist and a board-certified psychiatrist independently reviewed the diagnostic interview scoring notation, the medical examiner's report, any prior medical records, and a comprehensive narrative that summarized all scores of information about each subject. The social worker, the clinical psychologist, and the psychiatrist reached a consensus on the diagnosis. Cause of death was determined by the medical examiner. Subjects in either group who met DSM-IV criteria for substance use disorder (SUD) and/or major depressive disorder (MDD) diagnosis were included for this study. The presence of psychotropic medications and substances of abuse in blood and urine was determined by the medical examiner's office. Detailed subject demographic information is provided in Supplemental Tables 1-16. Information regarding potential confounding variables including time of death calculated as zeitgeber time (ZT), postmortem interval, tissue pH, race, age and sex, history of substance use, pharmacological treatment, sleep quality, suicide, duration of alcohol use disorder, duration of major depression, and mood symptom severity in the last two weeks of life were obtained from medical records, police reports, medical examiner's reports, and family interviews. Toxicology reports were used to determine the presence or absence of alcohol, cocaine, opioids, SSRIs, antidepressants, antipsychotics, lithium, and benzodiazepines in the blood at death. Exposure to psychiatric medications in the last month of life was estimated as described (Sullivan et al., 2018). Sleep quality was determined on subjective rating of chronic history of sleep

disturbances or history of excessive sleep reported in medical records and family interviews. Mood symptom severity in the last two weeks of life was determined from medical records and family interviews. Lifetime history of substance use was determined from medical records and family interviews and characterized as yes or no based on history of chronic use for each substance including alcohol, cocaine, opioids, and marijuana. Furthermore, subjective ratings of lifetime alcohol and nicotine intake were determined from medical records and family interviews and rated from 0 (none) to 4 (high). The presence or absence of recent history of calcium channel blockers was determined from medical records and family interviews. Anterior-posterior position of postmortem hippocampal samples was determined using the Atlas of the Human Brain by Jurgen K. Mai, Joseph Assheuer, and George Paxinos, Academic Press 1997.

Rhesus macaque subjects

Adult male rhesus macaques (n = 12 *Macaca mulatta,* (5 control and 7 alcohol) were trained to use operant drinking panels to obtain all fluids (water and/or alcohol) and meals. Once trained, they underwent 4 months of schedule-induced polydipsia to induce ethanol-self-administration in daily sessions. After SIP of water only, monkeys were induced to increasing volumes of 4% (w/v) ethanol in 30-day increments equivalent to 0.5, 1.0, and 1.5 g/kg/day, respectively. Following induction, “open access” (or 22 h/day concurrent access to ethanol on one spout and water on the other spout) began. The subjects received three meals (approximately 2 h apart) of banana pellets each session (day), with the first meal available at the session start. Each daily session of 22 h began at ZT5 and ended at ZT3 the following day. The lighting schedule in the room was 11-h light, 13-h dark, with lights turning off at ZT11. All subjects (control and ethanol drinking) Control subjects were housed in the same room. The self-administration protocols for both cohorts were the same, however a maltose dextrin solution (10% in water) was given to the controls to calorically match the drinkers and controls.

Immunofluorescence and Imaging

Paraformaldehyde Fixed Sections (Cohort B):

Sections containing the human hippocampus were carried through antigen retrieval in citric acid buffer (0.1 M citric acid, 0.2 M Na2HPO4) heated to 80 degrees °C for 30 minutes, incubated in 2% BSA for one hour. and incubated for two nights at 4 °C in biotinylated WFA lectin (catalog #B-1355, Vector Labs), PVB (catalog #P3088, Sigma-Aldrich), VAMP2 (catalog #10135-1-AP, ProteinTech), and SYN1 (catalog #20258-1-AP, ProteinTech) antibodies. Following primary antibody incubation, samples were incubated in alexa fluor streptavidin 488 (1:3000, cat# S11223, Invitrogen), goat anti-mouse 647 (1:300, cat#A28181), and goat anti-rabbit 555 (1:300; cat# A32732) for 4 hours, followed by incubation in Trueblack lipofuscin autofluorescence quencher (cat#23007, Biotum). Sections were mounted on gelatin coated glass slides and coverslipped with Dako fluorescent mounting media (cat#S3023, Agilent Technologies). A Zeiss LSM 880 confocal microscope interfaced with Zen imaging software (ZEN 2.3 SP1) was used to acquire images of PVB interneurons with WFA+ PNNs and VAMP2 or SYN1 labeling. Images were acquired with a z-step of 0.5 μm using a 63x oil immersion objective (numerical aperture 1.4 DIC M27; pixel size, 0.10 × .10 μm). Maximum intensity projections were acquired using Zen Blue (ZEN 2.6) software, compiling all images taken on the three-dimensional z-axis into a single, two-dimensional, multichannel image (Figure 2B-E and 2G-J).

Fresh Frozen Sections (Cohort A):

Sections containing the human hippocampus were post-fixed for 30 minutes in 4% paraformaldehyde, followed by 1-hour incubation in 2% bovine serum albumin (BSA), and overnight incubation in biotinylated *Wisteria floribunda agglutinin* (WFA) lectin (1:500, catalog #B-1355, Vector Labs) and mouse anti-GFAP (1:500, cat# 837201, Biolegend). The following day, samples were incubated in alexa fluor streptavidin 488 (1:3000, cat# S11223, Invitrogen) and goat anti-mouse 647 (1:300, cat#A28181) for 4 hours, followed by incubation in Trueblack lipofuscin autofluorescence quencher (cat#23007, Biotum). Sections were mounted on gelatin coated glass slides and

coverslipped with Dako fluorescent mounting media (cat#S3023, Agilent Technologies). An Olympus BX61 interfaced with Stereo-Investigator v11was used to acquire images WFA+ glia and GFAP labeling.

Quantitative polymerase chain reaction

RNA was extracted from 14 µm hippocampal cryosections using the RNeasy Minikit (cat# 74104, Qiagen, NL) according to the manufacturer's instructions. On-column DNAse digestion was carried out using the RNAse-free DNAse set (#79254, Qiagen, NL), per manufacturer's guide. Complementary DNA (cDNA) was synthesized using a High-Capacity cDNA Reverse Transcription Kit (Applied Biosystems, Foster City, CA, USA). For each reaction, 0.5 µl of cDNA (1:3 diluted) was placed in a 10 µL reaction containing 5 µL of SYBR Green PCR Master Mix (Applied Biosystems) and 10 pmol of each primer (Invitrogen, United States) or FastStart TaqMan Probe Master and 1x Taqman primers (ThermoFisher). The primers used are listed in Supplemental Table 2. Custom-made assays were tested for specificity and resulted in a single band of expected size. All reactions were performed in triplicate using 384-well optical reaction plates (Life Technologies, United States) on an Applied Biosystems detection system (QuantStudio 5, Applied Biosystems, Life Technologies, United States). Reactions were performed with an initial ramp time of 10 min at 95°C, and 40 subsequent cycles of 15 s at 95°C and 1 min at 60°C. For negative controls for the qPCR reactions, non-template control (cDNA was omitted) and no-RT control (reverse transcriptase excluded from cDNA synthesis reaction) were run on each plate. Relative concentrations of the transcripts of interest were calculated with comparison to a standard curve made with dilutions of cDNA from a pooled sampling of all the subjects. Values for the transcripts of interest were normalized to the geometric mean of B2M, ACTB, GAPDH and PPIA values for the same samples. Data were collected by QuantStudio Design and Analysis software v1.5.1.

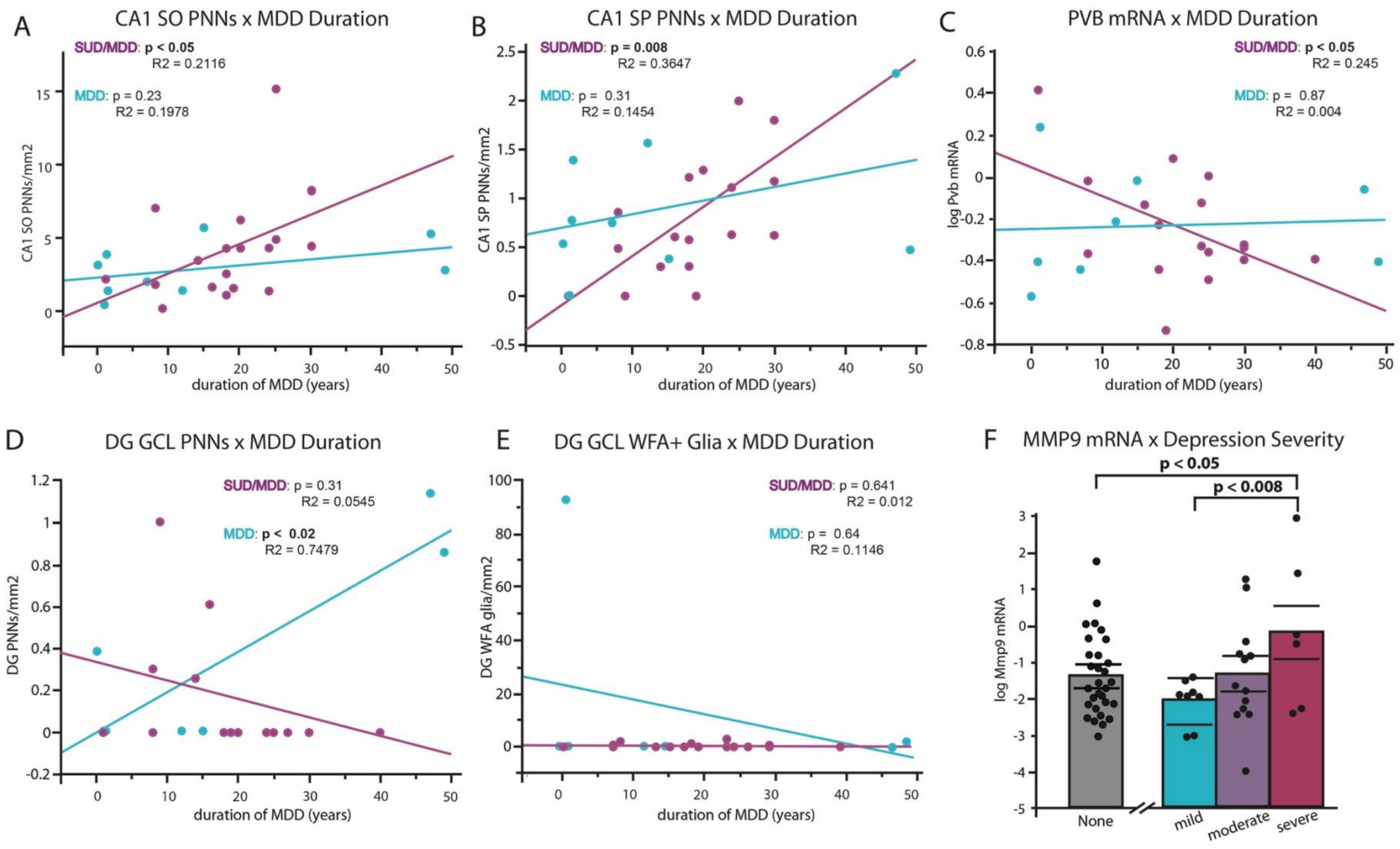

!"#$%&'()%*+",-!

Figure S1. Major depressive disorder duration and severity impacts ECM parameters in the human hippocampus. (A-E) PNNs in CA1 (A-B), bulk hippocampal PVB (C), and PNNs and WFA glia in the DG GCL plotted by the duration of MDD in subjects with MDD (blue) and MDD/SUD (purple). A significant positive correlation was observed in subjects with MDD/SUD in respect to CA1 PNNs in S.O. and S.P., while a negative

correlation was observed for PVB mRNA. Subjects with MDD, displayed a positive correlation between DG GCL PNNS and MDD duration, but no significant correlations were observed for any other parameters. (F) Subjects with MDD were placed into categories of depression severity.

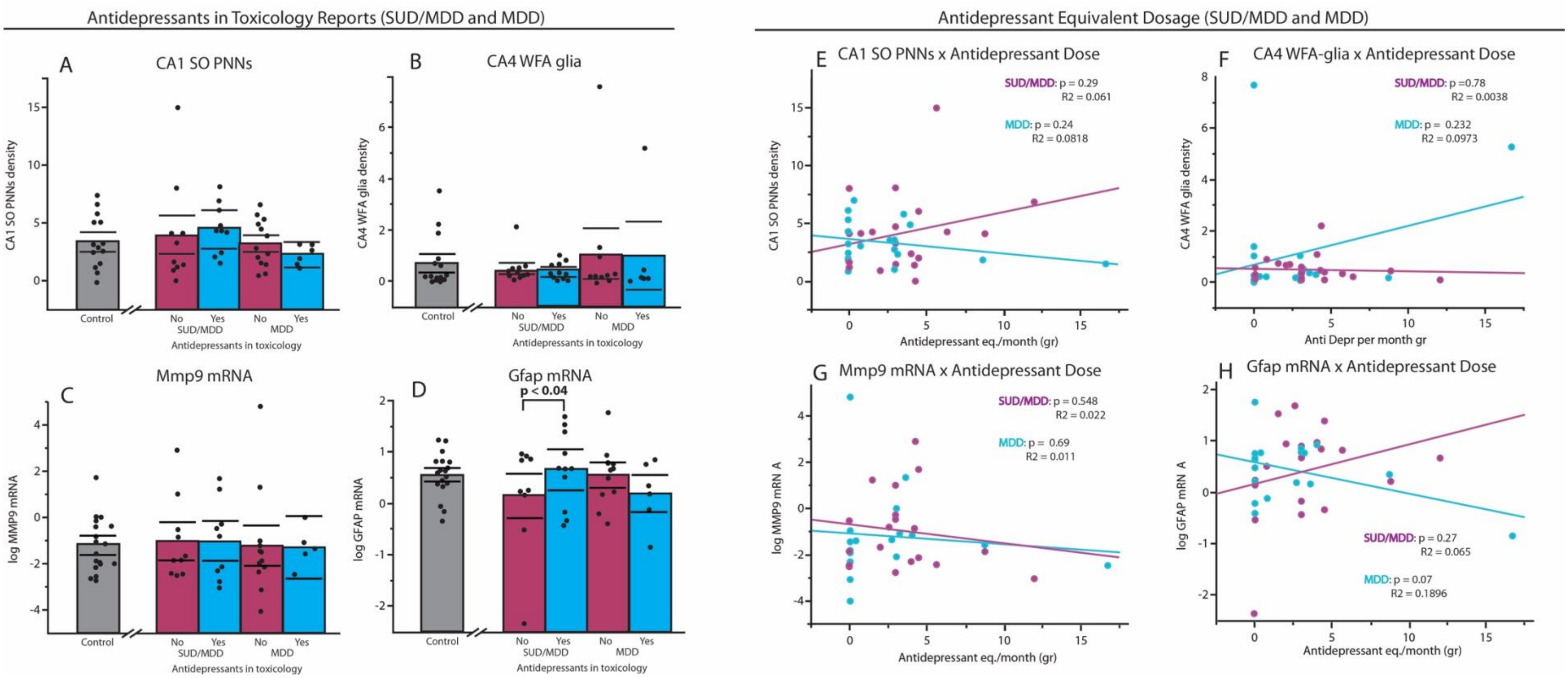

Figure S2. Effects of antidepressants on ECM parameters. No significant effects of antidepressants in the toxicology report were detected for CA1 SO PNNs (A), CA4 WFA+glia (B), or MMP9 mRNA (C). A significant increase in GFAP gene expression was observed in subjects with SUD/MDD with antidepressants in the toxicology report compared to SUD/MDD subjects without (D). Graphs represent mean of each group, black circles represent values for individual subjects, and black lines represent 95% confidence intervals. No significant correlations were detected in subjects with SUD/MDD and subjects with MDD for antidepressant equivalent amount per last month of life in grams with CA1 SO PNN densities, CA4 WFA-glia densities, MMP9 mRNA, or GFAP mRNA (E-H).

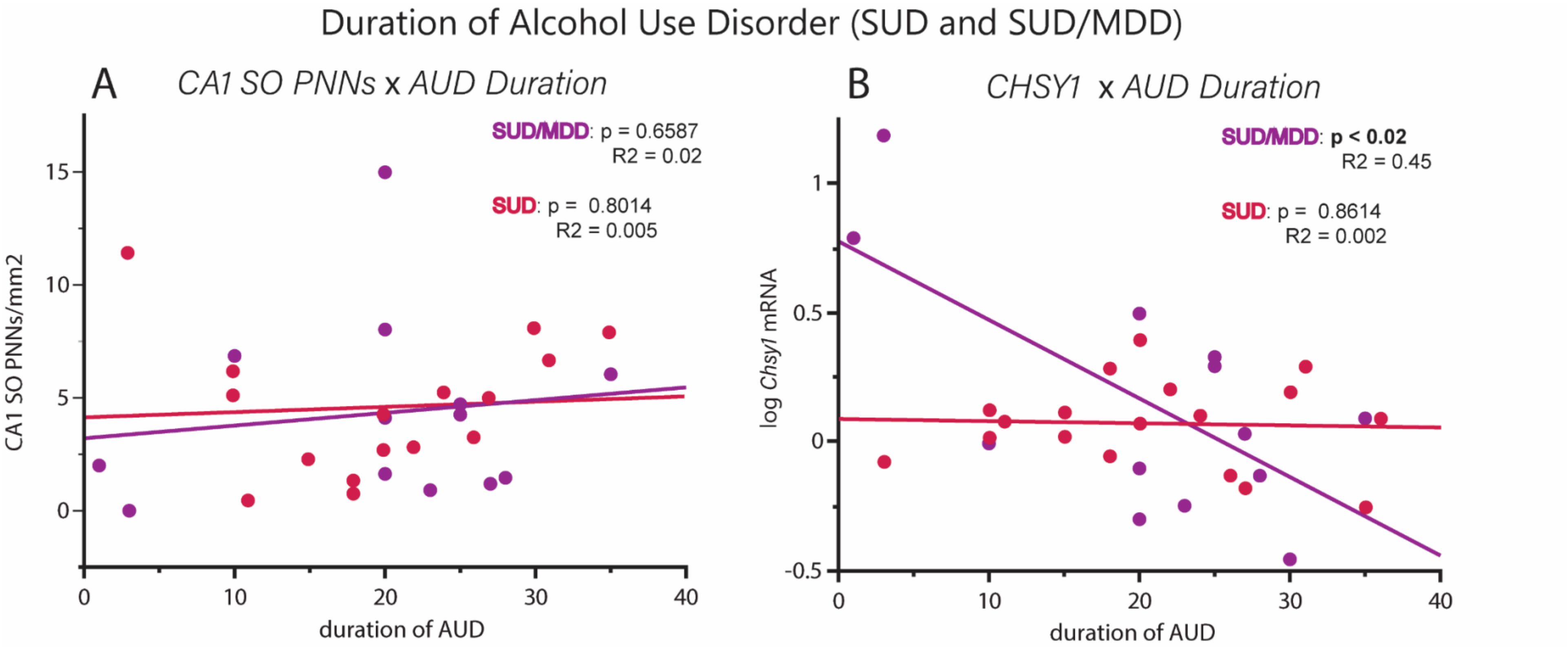

Figure S3. *Effect of AUD duration on CA1 SO PNNs and Chsy1 gene expression.* (A) No correlation was observed between CA1 SO PNNs and AUD duration in subjects with either SUD or SUD/MDD. (B) A negative correlation was observed between *Chsy1* gene expression and AUD duration in subjects with SUD/MDD. No correlation was observed in subjects with SUD.

Figure S4.

Effects of drugs in toxicology reports on ECM-related transcripts, PNNs and WFA⁺ glia. (A,B, and C) In subjects with SUD, cocaine in the toxicology report was associated with a decrease in *Pvb* and an increase in *Ctss* gene expression. No significant changes were observed for *Acan* mRNA. (D) Subjects with SUD and SUD/MDD who had a cocaine history had a lower density of PNNs compared to SUD and SUD/MDD subjects who did not have a cocaine use history. (E-H) Effects of alcohol in the toxicology report in control, SUD, SUD/MDD, and MDD subjects. No statistically significant effects were observed in any diagnostic group (Control and MDD groups did not have adequate numbers of subjects with ethanol in the toxicology report to perform statistical comparisons).

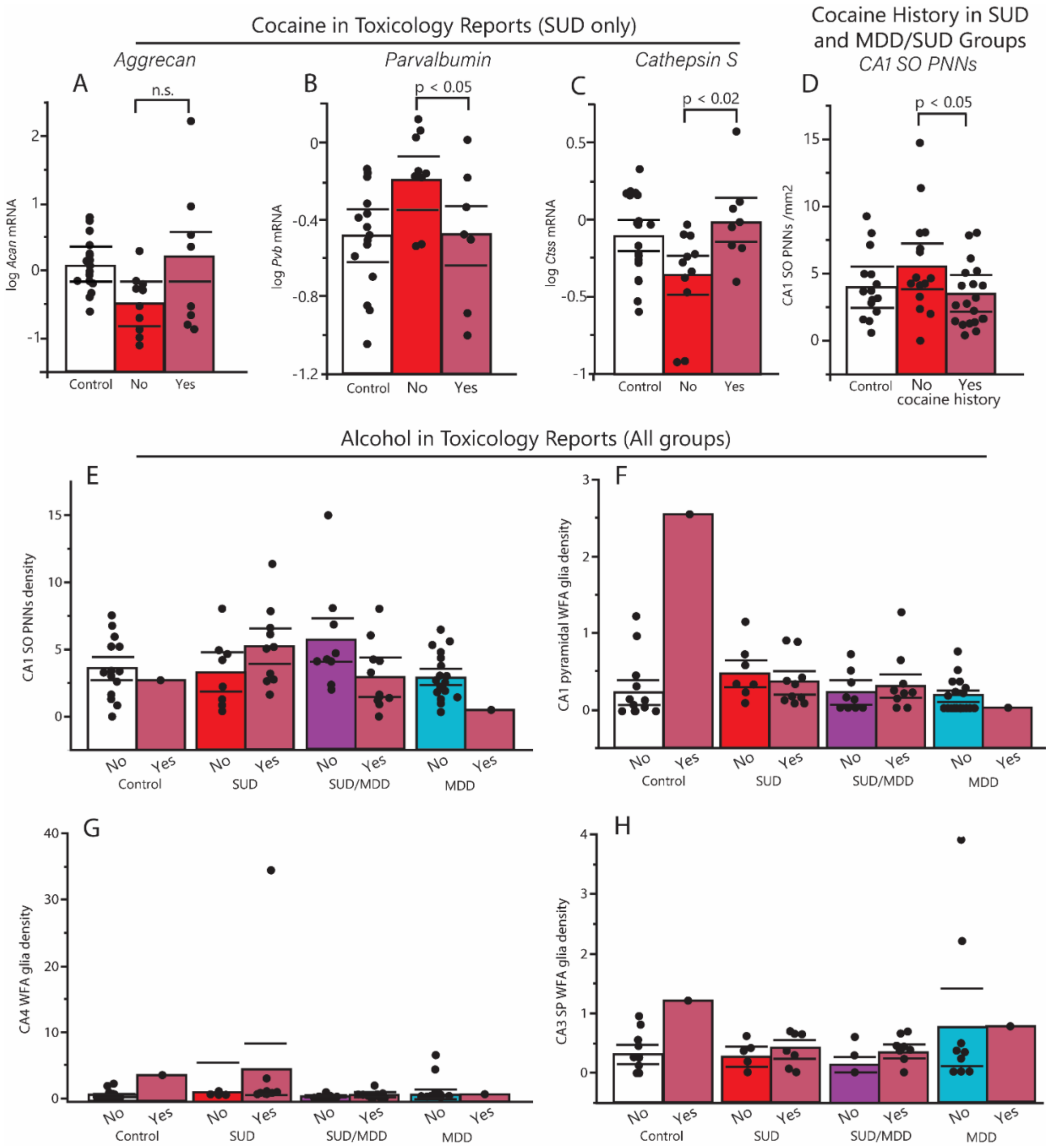

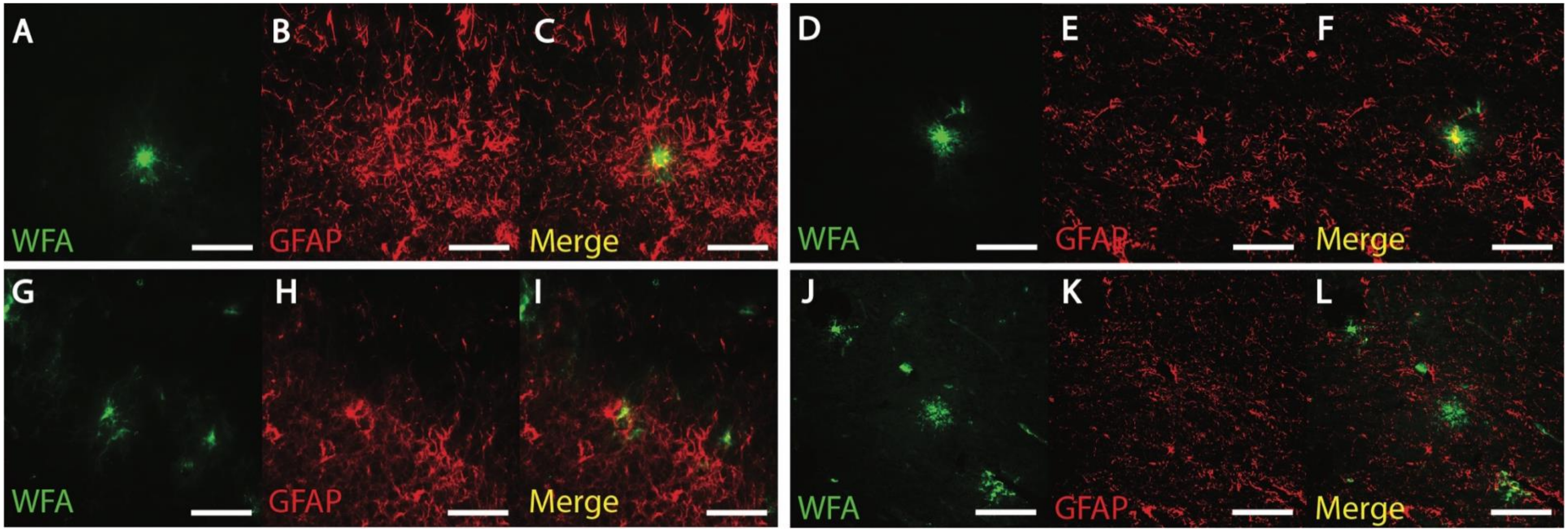

Figure S5. Multiplex immunofluorescence detects WFA labeling in GFAP+ astrocytes. (A-I) High-magnification images of WFA (A, D, and G), GFAP (B, E, H) and merged WFA/GFAP (C, F, I) demonstrates co-localization of WFA within astrocytes and their processes (yellow), scalebars = 50 μm. (J-L) Low magnification images depicting some WFA glial cells with lack of GFAP overlap, possibly reflecting fine processes of astrocytes with low GFAP levels, scalebar = 100 μm.

Table S1. Hippocampal Sub-Nuclei PNN Densities Across Diagnostic Groups

	Diagnosis		F Ratio	p value	Mean	Adj. least sq. mean	Standard error	Covariates
Dentate gyrus	**SUD**	↔	0.22	0.64	0.22	Con 0.15 ; SUD 0.22	Con 0.10 ; SUD 0.10	*None*
	**MDD**	↔	3.12	0.35	0.20	Con 0.20 ; MDD 0.07	Con 0.07 ; MDD 0.12	Duration of MDD
	**MDD/SUD**	↔	0.008	0.93	0.16	Con 0.15 ; MDD/SUD 0.16	Con 0.08 ; MDD/SUD 0.06	*None*
CA4	**SUD**	↑	**5.03**	**0.007**	0.75	Con -0.18 ; SUD 1.09	Con 0.28 ; SUD 0.22	Cocaine history
	**MDD**	↑	**5.42**	**0.01**	0.80	Con 0.06 ; MDD 0.67	Con 0.21 ; MDD 0.15	Anxiety disorder
	**MDD/SUD**	↔	0.36	0.55	0.64	Con 0.52 ; MDD/SUD 0.64	Con 0.16 ; MDD/SUD 0.13	*None*
CA3 Stratum Pyramidale	**SUD**	↔	0.83	0.37	0.54	Con 0.41 ; SUD 0.54	Con 0.10 ; SUD 0.09	*None*
	**MDD**	↔	3.18	0.86	0.37	Con 0.38 ; MDD 0.36	Con 0.10 ; MDD 0.10	PMI
	**MDD/SUD**	↑	**3.51**	**0.03**	0.65	Con 0.46 ; MDD/SUD 0.86	Con 0.13 ; MDD/SUD 0.14	Race
CA3 Stratum Oriens	**SUD**	↑	**3.85**	**0.01**	2.81	Con -0.21 ; SUD 4.84	Con 1.05 ; SUD 0.92	Cocaine history
	**MDD**	↔	4.76	0.066	2.43	Con 1.53 ; MDD 4.20	Con 0.62 ; MDD 0.98	Antidepressants/month, ZT time
	**MDD/SUD**	↔	0.004	0.95	2.23	Con 2.19 ; MDD/SUD 2.23	Con 0.55 ; MDD/SUD 0.47	*None*
CA2 Stratum Pyramidale	**SUD**	↑	**28.14**	**0.0001**	0.87	Con -2.05 ; SUD 2.81	Con 0.39 ; SUD 0.33	Cocaine history
	**MDD**	↔	2.79	0.06	0.97	Con -0.34 ; MDD 0.68	Con 0.46 ; MDD 0.36	*None*
	**MDD/SUD**	↑	**6.16**	**0.01**	1.24	Con -0.54 ; MDD/SUD 1.69	Con 0.59 ; MDD/SUD 0.39	SSRIs, ZT time
CA2 Stratum Oriens	**SUD**	↑	**3.67**	**0.03**	2.10	Con 0.22 ; SUD 4.57	Con 1.04 ; SUD 1.10	Age
	**MDD**	↔	3.41	0.10	3.16	Con 0.04 ; MDD 2.37	Con 1.18 ; MDD 0.94	SSRIs
	**MDD/SUD**	↔	0.16	0.69	2.03	Con 2.41 ; MDD/SUD 2.03	Con 0.73 ; MDD/SUD 0.61	*None*
CA1 Stratum Pyramidale	**SUD**	↔	1.56	0.16	0.94	Con 0.44 ; SUD 1.31	Con 0.39 ; SUD 0.30	*None*
	**MDD**	↓	**2.27**	**0.02**	0.91	Con 2.37 ; MDD 0.38	Con 0.55 ; MDD 0.36	*None*
	**MDD/SUD**	↓	**3.25**	**0.005**	0.84	Con 1.82 ; MDD/SUD -0.14	Con 0.35 ; MDD/SUD 0.37	Substance use history, duration of MDD
CA1 Stratum Oriens	**SUD**	↑	**5.79**	**0.007**	4.39	Con 1.47 ; SUD 5.65	Con 0.94 ; SUD 0.73	PMI and cocaine history
	**MDD**	↓	**2.87**	**0.05**	2.90	Con 1.90 ; MDD -0.09	Con 0.82 ; MDD 1.19	Sex, calcium channel blockers
	**MDD/SUD**	↔	4.72	0.29	4.19	Con 4.53 ; MDD/SUD 2.64	Con 1.12 ; MDD/SUD 0.86	EtOH in tox, duration of MDD

Values represent PNNs per mm^2

Table S2. Hippocampal Sub-Nuclei WFA-Glia Densities Across Diagnostic Groups

Values represent WFA-glia per mm^2

	Diagnosis		F Ratio	p value <	Mean	Adj. least sq. mean	Standard error	Covariates
Dentate gyrus	**SUD**	↑	4.24	**0.02**	1.02	Con -1.4 ; SUD 2.42	Con 0.93 ; SUD 0.74	Cocaine history
	**MDD**	↑	79.47	**0.0001**	5.90	Con -43.21 ; MDD 44.17	Con 3.40 ; MDD 3.10	Suicide, duration of MDD
	**MDD/SUD**	↓	4.16	**0.02**	0.36	Con 2.42 ; MDD/SUD 1.45	Con 0.59 ; MDD/SUD 0.60	EtOH in tox, calcium channel blockers
CA4	**SUD**	↑	6.04	**0.002**	2.59	Con -5.42 ; SUD 6.56	Con 2.33 ; SUD 1.84	Cocaine history
	**MDD**	↔	3.73	0.29	1.12	Con 0.69 ; MDD 0.35	Con 0.19 ; MDD 0.24	ZT time
	**MDD/SUD**	↓	3.86	**0.02**	0.40	Con 0.99 ; MDD/SUD 0.36	Con 0.20 ; MDD/SUD 0.14	EtOH in tox
CA3 Stratum Pyramidale	**SUD**	↔	4.57	0.09	0.32	Con 0.42 ; SUD 0.21	Con 0.09 ; SUD 0.08	Age, race
	**MDD**	↑	9.16	**0.0008**	0.75	Con -0.55 ; MDD 1.20	Con 0.31 ; MDD 0.23	Antidepressants in last month of life
	**MDD/SUD**	↓	6.16	**0.002**	0.25	Con 0.50 ; MDD/SUD 0.06	Con 0.09 ; MDD/SUD 0.09	EtOH in tox, race
CA3 Stratum Oriens	**SUD**	↔	2.70	0.45	0.39	Con 0.30 ; SUD 0.53	Con 0.21 ; SUD 0.20	Age
	**MDD**	↔	29.49	0.09	0.53	Con 1.84 ; MDD 1.58	Con 0.18 ; MDD 0.16	Antipsychotics, sex
	**MDD/SUD**	↔	0.61	0.44	0.31	Con 0.44 ; MDD/SUD 0.31	Con 0.13 ; MDD/SUD 0.11	*None*
CA2 Stratum Pyramidale	**SUD**	↔	8.95	0.73	0.52	Con 1.77 ; SUD 1.67	Con 0.40 ; SUD 0.34	Antidepressants in tox
	**MDD**	↑	5.88	**0.006**	1.76	Con -1.37 ; MDD 2.01	Con 0.78 ; MDD 0.59	Antidepressants in last month of life
	**MDD/SUD**	↓	5.26	**0.004**	0.00	Con 0.37 ; MDD/SUD -0.25	Con 0.11 ; MDD/SUD 0.15	Race
CA2 Stratum Oriens	**SUD**	↔	201.23	0.70	0.60	Con 2.48 ; SUD 2.53	Con 0.15 ; SUD 0.12	Antidepressants in tox
	**MDD**	↑	2.49	0.07	0.17	Con -0.18 ; MDD 0.26	Con 0.15 ; MDD 0.12	*None*
	**MDD/SUD**	↔	2.74	0.51	0.20	Con 0.23 ; MDD/SUD 0.16	Con 0.10 ; MDD/SUD 0.07	Cocaine history
CA1 Stratum Pyramidale	**SUD**	↔	0.17	0.69	0.32	Con 0.40 ; SUD 0.32	Con 0.14 ; SUD 0.13	*None*
	**MDD**	↔	2.22	0.15	0.15	Con 0.40 ; MDD 0.15	Con 0.13 ; MDD 0.11	*None*
	**MDD/SUD**	↓	3.65	**0.02**	0.25	Con 0.95 ; MDD/SUD 0.45	Con 0.22 ; MDD/SUD 0.15	Antipsychotics, EtOH in tox
CA1 Stratum Oriens	**SUD**	↔	4.10	0.75	0.32	Con 0.45 ; SUD 0.35	Con 0.22 ; SUD 0.20	Age, race
	**MDD**	↔	3.75	0.92	0.56	Con 0.62 ; MDD 0.58	Con 0.33 ; MDD 0.30	Tissue pH
	**MDD/SUD**	↓	3.97	**0.02**	0.14	Con 0.57 ; MDD/SUD -0.25	Con 0.21 ; MDD/SUD 0.25	Race

Table S3. Hippocampal Gene Expression Measurements Across Diagnostic Groups.

Values represent normalized gene expression.

	Diagnosis		F Ratio	p value	Adj. least sq. mean	Standard error	Covariates
Pvb	**SUD**	↑	**4.39**	**0.007**	Con -0.66 ; SUD -0.34	Con 0.10 ; SUD -0.07	Cocaine in tox
	**MDD**	↑	**9.33**	**0.004**	Con -0.52 ; MDD -0.24	Con 0.06 ; MDD 0.06	None
	**MDD/SUD**	↑	**7.33**	**0.002**	Con -0.67 ; MDD/SUD -0.10	Con 0.09 ; MDD 0.09	Duration of MDD
Mmp9	**SUD**	↓	**4.40**	**0.04**	Con -1.16 ; SUD -1.90	Con 0.25 ; SUD 0.25	None
	**MDD**	↔	4.49	0.14	Con -0.29 ; MDD -1.10	Con 0.47 ; MDD 0.34	Depression severity
	**MDD/SUD**	↔	2.54	0.53	Con -0.35 ; MDD/SUD -0.68	Con 0.50 ; MDD 0.37	Opioid dependence
Acan	**SUD**	↔	4.71	0.42	Con 0.11 ; SUD -0.10	Con 0.22 ; SUD 0.16	ZT time
	**MDD**	↔	3.85	0.15	Con -0.18 ; MDD -0.43	Con 0.12 ; MDD 0.12	Cocaine history
	**MDD/SUD**	↑	**3.06**	**0.03**	Con -0.45 ; MDD/SUD -0.01	Con 0.17 ; MDD/SUD 0.13	None
Chsy1	**SUD**	↑	**7.12**	**0.04**	Con -0.03 ; SUD 0.07	Con 0.03 ; SUD 0.03	Tissue pH
	**MDD**	↔	1.53	0.25	Con -0.04 ; MDD 0.004	Con 0.03 ; MDD 0.03	Tissue pH
	**MDD/SUD**	↑	**13.22**	**0.0001**	Con -0.25 ; MDD/SUD 0.48	Con 0.07 ; MDD/SUD 0.09	Duration of AUD, tissue pH
Gfap	**SUD**	↔	1.06	0.31	Con 0.56 ; SUD 0.12	Con 0.30 ; SUD 0.29	None
	**MDD**	↔	5.53	0.057	Con 0.64 ; MDD 0.34	Con 0.10 ; MDD 0.10	PMI, nicotine in tox
	**MDD/SUD**	↓	**4.35**	**0.02**	Con 1.01 ; MDD/SUD 0.24	Con 0.22 ; MDD/SUD 0.16	Antidepressant history
Vamp2	**SUD**	↑	**2.82**	**0.04**	Con -0.97 ; SUD -0.62	Con 0.07 ; SUD 0.11	Sleep quality
	**MDD**	↑	**9.03**	**0.005**	Con -0.97 ; MDD -0.74	Con 0.03 ; MDD 0.04	Age, ZT time, sleep disturbance
	**MDD/SUD**	↔	1.53	0.22	Con -0.85 ; MDD/SUD -0.76	Con 0.05 ; MDD/SUD 0.05	None
Syn1	**SUD**	↔	8.28	0.36	Con -0.03 ; SUD -0.09	Con 0.05 ; SUD 0.04	Ethanol in tox
	**MDD**	↔	2.21	0.09	Con -0.23 ; MDD -0.11	Con 0.06 ; MDD 0.04	SSRIs last month of life
	**MDD/SUD**	↔	3.48	0.89	Con -0.04 ; MDD/SUD -0.05	Con 0.06 ; MDD/SUD 0.04	SSRIs in tox
Ctss	**SUD**	↓	**3.61**	**0.03**	Con 0.05 ; SUD -0.26	Con 0.10 ; SUD 0.08	Cocaine in tox
	**MDD**	↑	**3.50**	**0.033**	Con -0.23 ; MDD 0.03	Con 0.06 ; MDD 0.09	History of alcohol use rating, ZT time
	**MDD/SUD**	↔	2.84	0.64	Con -0.39 ; MDD/SUD -0.33	Con 0.12 ; MDD/SUD 0.09	Cocaine history

Table S4. Hippocampal Area Measurements Across Diagnostic Groups.

Values represent areas in mm^2. SP: stratum pyramidale, SO: stratum oriens

**Human Subjects**							
	**Diagnosis**		**F Ratio**	**p value**	**Adj. least sq. mean**	**Standard error**	**Covariates**
Dentate gyrus	**SUD**	↔	0.002	0.96	Con 2.72 ; SUD 2.69	Con 0.46 ; SUD 0.44	*None*
	**MDD**	↔	0.07	0.80	Con 2.72 ; MDD 2.58	Con 0.46 ; MDD 0.40	None
	**MDD/SUD**	↔	0.02	0.89	Con 2.72 ; MDD/SUD 2.64	Con 0.46 ; MDD/SUD 0.39	*None*
CA4	**SUD**	↔	0.74	0.39	Con 14.38 ; SUD 19.20	Con 4.02 ; SUD 3.90	*None*
	**MDD**	↔	0.05	0.82	Con 14.38 ; MDD 13.57	Con 2.56 ; MDD 2.48	None
	**MDD/SUD**	↔	0.02	0.55	Con 14.38 ; MDD/SUD 13.96	Con 2.51 ; MDD/SUD 2.09	*None*
CA3 SP	**SUD**	↔	0.15	0.70	Con 5.60 ; SUD 6.12	Con 0.99 ; SUD 0.96	*None*
	**MDD**	↔	0.67	0.42	Con 5.60 ; MDD 4.73	Con 0.73 ; MDD 0.76	None
	**MDD/SUD**	↔	0.82	0.37	Con 5.60 ; MDD/SUD 4.58	Con 0.85 ; MDD/SUD 0.73	*None*
CA3 SO	**SUD**	↔	0.04	0.84	Con 2.30 ; SUD 2.41	Con 0.40 ; SUD 0.39	*None*
	**MDD**	↔	0.29	0.59	Con 2.30 ; MDD 2.03	Con 0.33 ; MDD 0.37	None
	**MDD/SUD**	↔	1.54	0.23	Con 2.30 ; MDD/SUD 1.70	Con 0.37 ; MDD/SUD 0.32	*None*
CA2 SP	**SUD**	↔	0.002	0.96	Con 1.31 ; SUD 1.29	Con 0.30 ; SUD 0.28	*None*
	**MDD**	↔	0.98	0.34	Con 1.31 ; MDD 1.67	Con 0.24 ; MDD 0.27	None
	**MDD/SUD**	↔	0.001	0.97	Con 1.31 ; MDD/SUD 1.30	Con 0.32 ; MDD/SUD 0.26	*None*
CA2 SO	**SUD**	↔	0.43	0.52	Con 0.74 ; SUD 0.91	Con 0.19 ; SUD 0.18	*None*
	**MDD**	↔	1.16	0.17	Con 0.74 ; MDD 1.07	Con 0.14 ; MDD 0.17	None
	**MDD/SUD**	↔	0.003	0.96	Con 0.74 ; MDD/SUD 0.73	Con 0.18 ; MDD/SUD 0.14	*None*
CA1 SP	**SUD**	↔	1.79	0.19	Con 7.01 ; SUD 10.40	Con 1.88 ; SUD 1.71	*None*
	**MDD**	↔	0.11	0.74	Con 7.01 ; MDD 7.51	Con 1.11 ; MDD 0.99	None
	**MDD/SUD**	↔	2.22	0.15	Con 7.01 ; MDD/SUD 10.90	Con 2.00 ; MDD/SUD 1.73	*None*
CA1 SO	**SUD**	↔	2.70	0.11	Con 3.10 ; SUD 4.42	Con 0.59 ; SUD 0.54	*None*
	**MDD**	↔	0.23	0.63	Con 3.10 ; MDD 3.40	Con 0.46 ; MDD 0.41	None
	**MDD/SUD**	↔	1.17	0.29	Con 3.10 ; MDD/SUD 4.42	Con 0.92 ; MDD/SUD 0.80	*None*
**Rhesus Monkeys**							
Dentate Gyrus	**Alcohol**	↔	0.08	0.79	Con 3.69 ; Alc 3.83	Con 0.38 ; Alc 0.32	N/A
CA4	**Alcohol**	↔	0.29	0.60	Con 7.90 ; Alc 9.00	Con 1.55 ; Alc 1.31	N/A
CA3 SP	**Alcohol**	↔	0.05	0.83	Con 4.31 ; Alc 4.47	Con 0.55 ; Alc 0.50	N/A
CA3 SO	**Alcohol**	↔	0.36	0.56	Con 2.96 ; Alc 3.84	Con 1.08 ; Alc 0.98	N/A
CA2 SP	**Alcohol**	↔	0.48	0.51	Con 1.58 ; Alc 1.98	Con 0.43 ; Alc 0.39	N/A
CA2 SO	**Alcohol**	↔	0.18	0.68	Con 1.50 ; Alc 1.39	Con 0.19 ; Alc 0.18	N/A
CA1 SP	**Alcohol**	↔	0.05	0.83	Con 14.60 ; Alc 14.94	Con 1.23 ; Alc 1.04	N/A
CA1 SO	**Alcohol**	↔	1.65	0.23	Con 8.56 ; Alc 7.01	Con 0.92 ; Alc 0.78	N/A

Tables S5-S8: Basic demographic information for all diagnosis groups

Table S5: Basic demographic information for subjects with substance use disorder

Age	Sex	Race	pH	PMI (hrs)	Sleep quality	ZT time	A-P Coordinates
			**SUBSTANCE USE DISORDER**				
40	M	Black	6.5	7	*NA*	-2.42	31.9
32	M	Black	6.82	14	*NA*	-6	26.5
29	M	White	6.75	27	*NA*	3.92	19.9
30	M	White	6.49	12	*NA*	-3.7	33.1
38	M	White	6.68	25	Decreased sleep	7.02	27.8
38	M	White	6.42	6	*NA*	*NA*	26.5
49	M	White	6.88	24	*NA*	*NA*	25.2
34	M	Black	6.5	16	*NA*	17.77	27.8
36	M	Black	6.97	38.5	Decreased sleep	-4.37	19.9
30	M	Black	6.8	21	*NA*	*NA*	23.9
42	M	White	6.86	30	*NA*	1.23	26.5
38	M	White	6.62	12	*NA*	17.03	39.5
31	F	White	6.18	12	Decreased sleep	*NA*	25.2
50	M	White	6.52	24	*NA*	*NA*	18.6
35	F	Black	6.43	26	*NA*	*NA*	16.0
22	F	White	6.12	12	*NA*	*NA*	21.2
56	M	Black	6.51	17	*NA*	13.14	33.1
28	M	White	6.3	26	Decreased sleep	3.67	21.2
52	M	White	6.81	8	*NA*	1.53	31.9
24	M	White	6.28	14	*NA*	*NA*	13.3
mean ± SD 34.8±9.0	3F, 17M	7B, 13W	6.57±0.23	18.58±8.5			25.5±6.5

Table S6: Basic demographic information for subjects with major depressive disorder

Age	Sex	Race	pH	PMI (hrs)	Sleep quality	ZT time	A-P Coordinates
			**MAJOR DEPRESSIVE DISORDER**				
43	M	White	6.73	21	Decreased sleep	6.25	18.6
63	F	White	6.3	18	Decreased sleep	10.53	27.8
42	M	White	6.64	20	*NA*	*NA*	22.6
50	F	White	6.83	23	Decreased sleep	6	27.8
34	F	White	6.27	24	Decreased sleep	NA	29.2
55	M	White	6.64	29	Decreased sleep	2.08	27.8
33	M	White	6.79	18	Increased sleep	*NA*	26.5
53	M	White	6.73	29	Decreased sleep	4.25	27.8
44	F	White	6.71	29	*NA*	2.83	23.9
59	M	White	6.22	27	Decreased sleep	8.47	23.9
46	M	Black	6.26	17	Decreased sleep	13.47	33.1
36	F	White	6.84	25	Increased sleep	7.12	27.8
35	M	White	6.96	11	Decreased sleep	11.33	21.2
41	M	White	6.24	19	Increased sleep	*NA*	23.9
56	F	White	5.61	38	Decreased sleep	*NA*	23.9
62	M	Black	6.06	22	Increased sleep	*NA*	17.2
20	M	White	6.73	20	Decreased sleep	*NA*	34.6
59	M	Black	6.6	31	Decreased sleep	*NA*	23.9
48	M	White	6.41	27	Normal sleep	2.78	31.9
61	M	White	6.74	25	Decreased sleep	6.33	26.6
mean ± SD 47±11.4	6F, 14M	3B, 17W	6.5±0.32	23.7±5.9			26.0±4.4

Table S7: Basic demographic information for subjects with comorbid substance use disorder and major depressive disorder

Age	Sex	Race	pH	PMI (hrs)	Sleep quality	ZT time	A-P Coordinates
			**COMORBID SUD AND MDD**				
36	M	White	6.72	15	Decreased sleep	15.33	19.9
37	M	White	6.89	19	*NA*	11.08	31.9
41	F	White	6.55	17	Decreased sleep	*NA*	25.2
47	F	White	6.65	9	*NA*	-3.5	27.8
48	F	White	6.13	24	Increased sleep	2.25	21.2
44	M	White	6.77	20	Decreased sleep	8.16	39.5
29	F	White	6.47	29	Decreased sleep	4.58	26.5
59	F	White	6.8	24	*NA*	6.68	34.6
40	M	White	6.66	26	*NA*	2.16	26.5
45	M	White	6.29	24	*NA*	*NA*	31.9
20	M	Black	6.21	10	Increased sleep	*NA*	22.6
35	M	White	6.81	24	*NA*	5.62	29.2
34	M	White	6.33	17	*NA*	13.25	14.6
37	M	White	6.59	18	*NA*	14	21.2
63	F	White	6.32	24	Decreased sleep	5.63	22.6
62	M	Black	6.52	17	Decreased sleep	15.98	23.9
54	M	White	6.54	38	Decreased sleep	8.5	17.2
48	F	Black	5.87	17	Decreased sleep	*NA*	21.2
43	M	White	6.6	20	Decreased sleep	13.65	21.2
42	F	White	6.84	12	Decreased sleep	-4.75	17.5
40	M	White	6.62	21	Normal sleep	8.5	25.2
58	M	Black	6.11	37	*NA*	*NA*	27.8
30	M	White	6.91	18	*NA*	12.42	34.6
62	M	White	6.7	5	*NA*	0.37	39.5
mean ± SD 43.9±11.1	8F, 16M	4B, 20W	6.54±0.27	20.2±7.6			26.0±6.7

Table S8: Basic demographic information for unaffected control subjects

Age	Sex	Race	pH	PMI (hrs)	Sleep quality	ZT time	A-P Coordinates
			**UNAFFECTED CONTROLS**				
62	F	White	6.34	27.5	Normal sleep	0.67	21.2
54	M	Black	6.53	19	Normal sleep	9.67	29.2
52	M	White	6.28	17	Normal sleep	13.15	23.9
30	M	Black	6.98	19	Normal sleep	13.92	27.8
48	M	Black	6.98	9	Normal sleep	0.28	18.6
51	F	Black	6.3	22	Normal sleep	8.25	23.9
49	F	Black	6.57	29	Normal sleep	5	22.6
38	F	White	5.93	13	Normal sleep	-1.88	23.9
44	F	Black	6.72	32	Decreased sleep	2.17	23.9
44	M	White	6.6	24.32	Normal sleep	*NA*	39.5
28	M	Black	6.32	35.3	Normal sleep	-2.22	17.2
42	F	White	6.6	21	Normal sleep	10.72	23.9
31	M	White	6.78	14.15	Normal sleep	15	29.2
51	M	White	6.76	17	Decreased sleep	16.17	10.7
29	F	Black	6.64	25.5	Normal sleep	*NA*	39.5
35	M	Black	6.26	21	Normal sleep	9.5	21.2
49	M	White	6.71	9.75	Decreased sleep	-1.67	27.8
17	M	White	6.66	22.75	Normal sleep	-4.27	31.9
51	M	White	6.3		Normal sleep	*NA*	30.5
59	M	White	6.47	23.75	Normal sleep	6.75	23.9
mean ± SD 43.2±11.42	7F, 13M	9B, 11W	6.5±0.25	21.2±6.8			25.5±6.9

Tables S9-S11: Substance use information for all diagnostic groups

Table S9: Substance use information for subjects with substance use disorder

case	Onset of AUD (age)	Duration of AUD (yrs)	Ethanol in toxicology report	Cocaine in toxicology report	Opioids in toxicology report	Opioid dependence	Smoker	Nicotine rating	Alcohol rating	Cannabis history	Drug abuse type
**SUBSTANCE USE DISORDER**											
40M	14	26	Yes	Yes	No	No	Yes	2	4	No	Polysubstance abuse
32M	*NA*	*NA*	Yes	Yes	No	No	Yes	3	2	Yes	Polysubstance abuse
29M	14	15	No	No	No	No	Yes	4	4	Yes	Polysubstance abuse
30M	20	10	Yes	No	No	No	Yes	*NA*	4	Yes	Polysubstance abuse
38M	20	18	No	No	Yes	Yes	No	0	2	Yes	Polysubstance abuse
38M	16	22	Yes	No	Yes	Yes	Yes	3	4	Yes	Polysubstance abuse
49M	18	31	Yes	No	No	Yes	Yes	4	4	Yes	Polysubstance abuse
34M	16	18	No	Yes	No	No	Yes	4	4	Yes	Polysubstance abuse
36M	16	20	Yes	No	No	No	Yes	2	4	Yes	Polysubstance abuse
30M	19	11	No	Yes	No	No	Yes	2	3	Yes	Polysubstance abuse
42M	15	27	No	No	No	No	Yes	4	4	No	Alcohol abuse
38M	14	24	Yes	Yes	Yes	Yes	Yes	4	4	Yes	Polysubstance abuse
31F	16	15	No	No	No	No	Yes	4	4	Yes	Polysubstance abuse
50M	14	36	Yes	Yes	No	Yes	No	0	4	No	Polysubstance abuse
35F	15	20	No	Yes	No	No	No	0	4	Yes	Polysubstance abuse
22F	12	10	Yes	No	No	No	Yes	4	4	Yes	Polysubstance abuse
56M	26	30	No	Yes	No	No	No	0	4	Yes	Polysubstance abuse
28M	*NA*	*NA*	No	No	Yes	Yes	Yes	2	4	Yes	Polysubstance abuse
52M	16	35	Yes	No	No	No	Yes	4	4	Yes	Polysubstance abuse
24M	21	3	Yes	Yes	Yes	Yes	Yes	4	4	Yes	Polysubstance abuse
mean ± SD 34.8±9.0/ 3F, 17M											

Table S10: Substance use information for subjects with major depressive disorder

case	Onset of AUD (age)	Duration of AUD (yrs)	Ethanol in toxicology report	Cocaine in toxicology report	Opioids in toxicology report	Opioid dependence	Smoker	Nicotine rating	Alcohol rating	Cannabis history	Drug abuse type
**MAJOR DEPRESSIVE DISORDER**											
43M	None	0	No	No	No	No	No	0	0	No	None
63F	None	0	No	No	No	No	Yes	4	1	No	None
42M	None	0	No	No	No	No	No	0	0	No	None
50F	None	0	No	No	No	No	Yes	4	1	No	None
34F	None	0	No	No	No	No	No	0	0	No	None
55M	None	0	No	No	No	No	Yes	3	0	No	None
33M	None	0	No	No	No	No	No	0	1	No	None
53M	None	0	No	No	No	No	No	0	1	No	None
44F	None	0	No	No	No	No	Yes	3	0	No	None
59M	None	0	No	No	No	No	Yes	4	2	No	None
46M	None	0	No	No	No	No	No	0	2	No	None
36F	None	0	No	No	No	No	Yes	4	2	No	None
35M	None	0	No	No	No	No	No	0	1	No	None
41M	None	0	No	No	No	No	Yes	4	1	No	None
56F	None	0	No	No	No	No	Yes	4	2	No	None
62M	None	0	No	No	No	No	Yes	3	1	No	None
20M	None	0	No	No	No	No	No	0	1	No	None
59M	None	0	No	No	No	No	No	0	1	No	None
48M	None	0	No	No	No	No	Yes	3	1	No	None
61M	None	0	No	No	Yes	No	Yes	2	1	No	None
mean ± SD 47±11.4/ 6F, 14M											

Table S11: Substance use information for subjects with comorbid substance use disorder and major depressive disorder

case	Onset of AUD (age)	Duration of AUD (yrs)	Ethanol in toxicology report	Cocaine in toxicology report	Opioids in toxicology report	Opioid dependence	Smoker	Nicotine rating	Alcohol rating	Cannabis history	Drug abuse type
**COMORBID SUD AND MDD**											
36M	8	28	Yes	Yes	No	No	Yes	4	4	Yes	Polysubstance abuse
37M	14	23	Yes	No	No	No	Yes	4	4	Yes	Polysubstance abuse
41F	*NA*	*NA*	Yes	No	No	Yes	No	*NA*	1	No	Polysubstance abuse
47F	27	20	No	No	No	Yes	Yes	4	4	Yes	Polysubstance abuse
48F	45	3	Yes	No	No	No	Yes	4	4	No	Polysubstance abuse
44M	34	10	No	No	No	No	Yes	4	4	No	Polysubstance abuse
29F	*NA*	*NA*	No	No	Yes	Yes	Yes	4	4	No	Polysubstance abuse
59F	34	25	No	No	Yes	No	Yes	4	4	No	Polysubstance abuse
40M	13	27	Yes	Yes	No	No	Yes	*NA*	4	Yes	Polysubstance abuse
45M	*NA*	*NA*	Yes	No	No	No	Yes	4	4	No	Polysubstance abuse
20M	19	1	No	No	No	No	No	0	3	Yes	Polysubstance abuse
35M	*NA*	*NA*	No	No	No	No	No	0	4	No	Polysubstance abuse
34M	14	20	Yes	No	No	No	Yes	2	3	Yes	Polysubstance abuse
37M	14	23	Yes	No	Yes	Yes	Yes	3	4	No	Polysubstance abuse
63F	38	25	Yes	No	No	Yes	Yes	4	4	No	Polysubstance abuse
62M	42	20	Yes	No	No	No	Yes	2	4	No	Polysubstance abuse
54M	24	30	No	No	No	No	Yes	4	4	No	Polysubstance abuse
48F	*NA*	*NA*	Yes	No	Yes	Yes	Yes	3	4	Yes	Polysubstance abuse
43M	*NA*	*NA*	Yes	No	No	Yes	Yes	4	1	No	Polysubstance abuse
42F	*NA*	*NA*	No	No	No	Yes	Yes	1	3	Yes	Polysubstance abuse
40M	*NA*	*NA*	No	No	No	No	Yes	4	2	Yes	Polysubstance abuse
58M	23	35	Yes	No	Yes	Yes	Yes	4	4	No	Polysubstance abuse
30M	*NA*	*NA*	Yes	No	No	No	Yes	3	3	Yes	Alcohol abuse
62M	*NA*	20	No	No	No	No	Yes	4	4	No	*NA*
mean ± SD 43.9±11.1/ 8F, 16M											

Table S12: Substance use information for unaffected control subjects

case	Onset of AUD (age)	Duration of AUD (yrs)	Ethanol in toxicology report	Cocaine in toxicology report	Opioids in toxicology report	Opioid dependenc e	Smoker	Nicotine rating	Alcohol rating	Cannabis history	Drug abuse type
						**UNAFFECTED CONTROLS**					
62F	None	0	No	No	No	No	No	0	1	No	None
54M	None	0	No	No	No	No	No	4	3	No	None
52M	None	0	No	No	No	No	No	0	0	No	None
30M	None	0	No	No	No	No	No	1	1	No	None
48M	None	0	No	No	No	No	No	4	1	No	None
51F	None	0	No	No	No	No	Yes	4	1	Yes	Cannabis
49F	None	0	No	No	No	No	No	0	3	No	None
38F	None	0	No	No	No	No	No	0	0	No	None
44F	None	0	No	No	No	No	Yes	2	1	No	None
44M	None	0	No	No	No	No	Yes	4	1	No	None
28M	None	0	No	No	No	No	No	0	1	No	None
42F	None	0	No	No	No	No	Yes	4	2	Yes	Cannabis
31M	None	0	No	No	No	No	Yes	4	2	No	None
51M	None	0	No	No	No	No	Yes	4	2	No	None
29F	None	0	No	No	No	No	Yes	2	2	Yes	Cannabis
35M	None	0	No	No	No	No	Yes	2	2	No	None
49M	None	0	No	No	No	No	Yes	4	2	Yes	Cannabis
17M	None	0	Yes	No	No	No	No	0	2	No	None
51M	None	0	No	No	No	No	No	1	2	Yes	Cannabis
59M	None	0	No	No	No	No	Yes	3	1	No	None
mean ± SD 43.2±11.42/ 7F, 13M											

Tables S13-S16: Mood disorder-related information for all diagnostic groups

Table S13: Mood disorder-related information for subjects with substance use disorders

case	Suicide	Depression severity	Duration of MDD (yrs.)	Antidepressants in blood at death	Antidepressants/ month (grams)	Mania	Psychosis	Antipsychotics
**SUBSTANCE USE DISORDERS**								
62F	No	None	0	No	0	No	No	No
54M	No	None	0	No	0	No	No	No
52M	No	Mild	0	No	0	No	No	No
30M	No	None	0	No	0	No	No	No
48M	No	*NA*	0	Yes	4.5	No	No	No
51F	No	None	0	No	0	No	No	No
49F	Yes	None	0	No	0	No	No	No
38F	No	None	0	No	0	No	No	No
44F	No	None	0	No	0	No	No	No
44M	No	None	0	No	0	No	No	No
28M	No	None	0	No	0	No	No	No
42F	No	None	0	No	0	No	No	No
31M	No	*NA*	*NA*	Yes	10.2	No	Yes	Yes
51M	Yes	*NA*	*NA*	Yes	4.8	No	No	No
29F	No	None	*NA*	No	0	No	No	No
35M	No	None	0	No	0	No	No	No
49M	No	None	0	No	0	No	No	No
17M	No	None	0	No	0	No	No	No
51M	No	*NA*	*NA*	No	0	No	No	No
59M	Yes	*NA*	*NA*	Yes	2.6	No	No	No
mean ± SD 34.8±9.0/ 3F, 17M								

Table S14: Mood disorder-related information for subjects with major depressive disorder

case	Suicide	Depression severity	Duration of MDD (yrs.)	Antidepressants in blood at death	Antidepressants/ month (grams)	Mania	Psychosis	Antipsychotics
**MAJOR DEPRESSIVE DISORDER**								
43M	Yes	Moderate	15	No	0	No	Yes	No
63F	No	Moderate	*NA*	Yes	3	No	No	No
42M	Yes	Severe	*NA*	Yes	0.8	No	No	No
50F	Yes	Moderate	*NA*	Yes	0	No	Yes	No
34F	Yes	Severe	*NA*	Yes	3	No	No	Yes
55M	Yes	*NA*	*NA*	Yes	3.2	No	No	No
33M	Yes	Moderate	*NA*	Yes	16.7	No	No	Yes
53M	Yes	*NA*	*NA*	Yes	4	No	No	No
44F	Yes	*NA*	12	Yes	8.7	No	No	No
59M	Yes	*NA*	0.08	Yes	3	No	No	No
46M	No	Mild	1	No	0	No	No	No
36F	No	Mild	*NA*	No	0	No	No	No
35M	No	Moderate	1.5	No	0	No	No	No
41M	No	Moderate	7	No	0	No	No	No
56F	No	*NA*	1	No	0	No	No	No
62M	No	Mild	49	No	0	No	No	No
20M	Yes	Moderate	1.33	No	0	No	No	No
59M	Yes	*NA*	*NA*	Yes	0.4	No	No	No
48M	No	*NA*	*NA*	Yes	2.7	No	No	No
61M	Yes	Severe	47	Yes	3.6	No	No	No
mean ± SD 47±11.4/ 6F, 14M								

Table S15: Mood disorder-related information for subjects with comorbid substance use disorder and major depressive disorder

case	Suicide	Depression severity	Duration of MDD (yrs.)	Antidepressants in blood at death	Antidepressants/ month (grams)	Mania	Psychosis	Antipsychotics
**COMORBID SUD AND MDD**								
36M	Yes	Severe	16	Yes	3	No	Yes	No
37M	Yes	Moderate	18	Yes	2	No	No	No
41F	Yes	Moderate	14	Yes	3	No	No	No
47F	No	Moderate	20	Yes	0	No	No	No
48F	Yes	Severe	9	Yes	4.3	No	No	No
44M	Yes	Mild	8	Yes	12	No	No	No
29F	No	Severe	18	Yes	4	No	Yes	No
59F	Yes	Moderate	25	Yes	3	No	No	No
40M	Yes	*NA*	24	No	0	No	No	No
45M	Yes	Moderate	25	Yes	2.6	No	No	No
20M	Yes	*NA*	1	Yes	4.5	No	No	No
35M	Yes	*NA*	18	Yes	8.8	No	No	Yes
34M	Yes	Mild	8	No	0	No	No	No
37M	Yes	Severe	27	Yes	2.3	No	Yes	Yes
63F	No	Moderate	30	Yes	1.5	No	Yes	Yes
62M	No	Severe	30	No	0	No	No	No
54M	No	*NA*	40	Yes	3	No	No	No
48F	No	Moderate	19	Yes	4.2	No	No	No
43M	Yes	Moderate	24	Yes	0.8	No	No	No
42F	No	Moderate	*NA*	Yes	6.4	No	Yes	Yes
40M	No	Severe	30	Yes	3	No	Yes	No
58M	No	*NA*	20	Yes	4.5	No	No	No
30M	Yes	Moderate	*NA*	No	0	No	No	No
62M	Yes	Severe	25	Yes	5.7	No	No	No
mean ± SD 43.9±11.1/ 8F, 16M								

Table S16: Mood disorder-related information for unaffected control subjects

case	Suicide	Depression severity	Duration of MDD (yrs.)	Antidepressants in blood at death	Antidepressants/ month (grams)	Mania	Psychosis	Antipsychotics
**UNAFFECTED CONTROLS**								
62F	No	None	0	No	0	No	No	No
54M	No	None	0	No	0	No	No	No
52M	No	None	0	No	0	No	No	No
30M	No	None	0	No	0	No	No	No
48M	No	None	0	No	0	No	No	No
51F	No	None	0	No	0	No	No	No
49F	No	None	0	No	0	No	No	No
38F	No	None	0	No	0	No	No	No
44F	No	Mild	0	No	0	No	No	No
44M	No	None	0	No	0	No	No	No
28M	No	None	0	No	0	No	No	No
42F	No	Mild	0	No	0	No	No	No
31M	No	None	0	No	0	No	No	No
51M	No	None	0	No	0	No	No	No
29F	No	Mild	0	No	0	No	No	No
35M	No	None	0	No	0	No	No	No
49M	No	None	0	No	0	No	No	No
17M	No	None	0	No	0	No	No	No
51M	No	None	0	No	0	No	No	No
59M	No	None	0	No	0	No	No	No
mean ± SD 43.2±11.42/ 7F, 13M								

Tables S17-S20: Other relevant demographic information for all diagnostic groups

Table S17: Other relevant information for subjects with substance use disorders

case	Anxiety disorders	Personality disorders	Obsessive compulsive disorder	Lithium	Lithium/ mo (grams)	Calcium channel blockers	Antipsychotics last month of life (CPZ eq./grams)	Valproic acid last month of life (grams)
**SUBSTANCE USE DISORDERS**								
62F	None	Antisocial	No	None	0	No	0	0
54M	None	Antisocial	No	None	0	No	0	0
52M	None	Antisocial	No	None	0	No	0	0
30M	None	None	No	None	0	No	0	0
48M	None	None	No	None	0	No	0	0
51F	None	Antisocial	No	None	0	No	0	0
49F	None	Antisocial	No	None	0	No	0	0
38F	None	Antisocial	No	None	0	No	0	0
44F	None	Antisocial	No	None	0	No	0	0
44M	None	None	No	None	0	No	0	0
28M	None	None	No	None	0	No	0	0
42F	PTSD	Antisocial	No	None	0	No	0	0
31M	GAD	Borderline/ Antisocial	No	None	0	No	6	0
51M		Antisocial	No	None	0	No	0	0
29F	None	None	No	None	0	No	0	0
35M	None	None	No	None	0	No	0	0
49M	None	None	No	None	0	No	0	0
17M	None	None	No	None	0	No	0	0
51M	None	Antisocial	No	None	0	No	0	0
59M	None	Borderline	No	None	0	No	0	0
mean ± SD 34.8±9.0/ 3F, 17M								

Table S18: Other relevant information for subjects with major depressive disorder

case	Anxiety disorders	Personality disorders	Obsessive compulsive disorder	Lithium	Lithium/ mo (grams)	Calcium channel blockers	Antipsychotics last month of life (CPZ eq./grams)	Valproic acid last month of life (grams)
**MAJOR DEPRESSIVE DISORDER**								
43M	None	None	Yes	None	0	No	0	0
63F	None	Schizoid	No	None	0	No	0	0
42M	None	None	No	None	0	No	0	0
50F	None	None	No	None	0	No	0	0
34F	Panic	None	No	None	0	No	1.1	7.5
55M	None	None	No	None	0	No	0	0
33M	None	Dependent	No	Yes	12	No	6.8	0
53M	None	None	No	None	0	*NA*	0	0
44F	None	None	No	None	0	No	0	0
59M	GAD	None	No	None	0	No	0	0
46M	None	Schizoid	No	None	0	No	0	0
36F	Panic	None	No	None	0	Yes	0	0
35M	None	None	Yes	None	0	No	0	0
41M	None	None	No	None	0	No	0	0
56F	GAD	None	No	None	0	No	0	0
62M	None	None	No	None	0	Yes	0	0
20M	Adjustment	None	No	None	0	No	0	0
59M	None	None	No	None	0	No	0	0
48M	*NA*	*NA*	*NA*	None	0	No	0	0
61M	None	None	No	None	0	No	0	0
mean ± SD 47±11.4/ 6F, 14M								

Table S19: Other relevant information for subjects with comorbid substance use disorder and major depressive disorder

case	Anxiety disorders	Personality disorders	Obsessive compulsive disorder	Lithium	Lithium/ mo (grams)	Calcium channel blockers	Antipsychotics last month of life (CPZ eq.)	Valproic acid last month of life (grams)
				**COMORBID SUD AND MDD**				
36M	None	None	No	None	0	No	0	0
37M	None	Dependent	No	None	0	No	0	0
41F	None	None	No	None	0	No	0	0
47F	Phobia	Histrionic	No	None	0	No	0	0
48F	None	None	No	None	0	No	0	0
44M	PTSD	None	No	None	0	No	0	0
29F	None	Borderline	No	None	0	No	0	0
59F	Panic	None	No	None	0	No	0	0
40M	None	None	No	None	0	No	0	0
45M	None	Borderline	No	None	0	No	0	0
20M	GAD	None	No	None	0	No	0	0
35M	None	Mixed	No	None	0	No	2	0
34M	None	Borderline	No	None	0	No	0	0
37M	None	Borderline / Antisocial	No	None	0	No	1.8	0
63F	None	Dependent	No	None	0	No	0.9	0
62M	None	Borderline	No	None	0	No	0	0
54M	None	None	No	None	0	No	0	0
48F	None	None	No	None	0	No	0	0
43M	None	None	No	None	0	No	0	0
42F	None	None	No	None	0	No	3.6	0
40M	None	Dependent	No	None	0	No	0	0
58M	None	None	No	None	0	No	0	0
30M	None	Borderline	No	None	0	No	0	0
62M	*NA*	*NA*	*NA*	None	0	No	0	0
mean ± SD 43.9±11.1/ 8F, 16M								

Table S20: Other relevant information for unaffected control subjects

case	Anxiety disorders	Personality disorders	Obsessive compulsive disorder	Lithium	Lithium/ mo (grams)	Calcium channel blockers	Antipsychotics last month of life (CPZ eq.)	Valproic acid last month of life (grams)
**UNAFFECTED CONTROLS**								
62F	*NA*	*NA*	*NA*	None	0	No	0	0
54M	None	None	Yes	None	0	No	0	0
52M	*NA*	*NA*	*NA*	None	0	No	0	0
30M	None	None	No	None	0	No	0	0
48M	None	None	No	None	0	No	0	0
51F	None	None	Yes	None	0	No	0	0
49F	*NA*	*NA*	*NA*	None	0	No	0	0
38F	*NA*	*NA*	*NA*	None	0	No	0	0
44F	None	None	Yes	None	0	*NA*	0	0
44M	None	None	Yes	None	0	No	0	0
28M	None	None	No	None	0	No	0	0
42F	None	None	Yes	None	0	No	0	0
31M	*NA*	*NA*	*NA*	None	0	No	0	0
51M	*NA*	*NA*	*NA*	None	0	No	0	0
29F	None	None	No	None	0	No	0	0
35M	None	None	No	None	0	Yes	0	0
49M	None	None	No	None	0	No	0	0
17M	*NA*	*NA*	*NA*	None	0	No	0	0
51M	None	Paranoid	No	None	0	No	0	0
59M	None	None	Yes	None	0	No	0	0
mean ± SD 43.2±11.42/ 7F, 13M								

***NA* = information not available**
GAD = generalized anxiety disorder
PTSD = post-traumatic stress disorder

Table S21. Primers and reagents

Probes	Forward	Reverse
**EXPERIMENTAL**		
Pvb	GCTACCGACTCCTTCGAC	ATGAATCCCAGCTCATCC
Gfap	TaqMan: Hs00909236_m1	
Vamp2	CATGAGGGTGAACGTGGACA	TGCGCTTGTTTCAAACTGGG
Syn1	GTGGACACGTGCTCAGAGAT	AGGAACCCACCACCTCAATG
Mmp9	TaqMan: Hs00234579_m1	
Ctss	ACAAGGGCATCGACTCAGAC	TTTGAACATGTGGCAGCACG
Acan	TaqMan: Hs00153936_m1	
Chsy1	GTGGCCGCCTACAGAACAT	AGTGGCACTACTGGAATTGGT
**LOADING CONTROLS**		
Actb	GTCATTCCAAATATGAGATGCGT	GCTATCACCTCCCCTGTGTG
Ppia	ATGGTCAACCCCACCGTGTTCTTCG	CGTGTGAAGTCACCACCCTGACACA
Gapdh	TCGACAGTCAGCCGCATCT	AGTTAAAAGCAGCCCTGGTGA
B2m	GTGGGATCGAGACATGTAAGC	AGCAAGCAAGCAGAATTTGGAAT

1. Zhu H, Urban DJ, Blashka J, McPheeters MT, Kroeze WK, Mieczkowski P, et al. Quantitative analysis of focused a-to-I RNA editing sites by ultra-high-throughput sequencing in psychiatric disorders. PLoS One. 2012;7(8):e43227. Epub 2012/08/23. doi: 10.1371/journal.pone.0043227. PubMed PMID: 22912834; PubMed Central PMCID: PMC3422315.
